# Supplementary material for: Liquid spherical shells are a non-equilibrium steady state of active droplets
Source: Nat Commun. 2023 Oct 17;14:6552. doi: 10.1038/s41467-023-42344-w (PMC10582082; doi:10.1038/s41467-023-42344-w)
Supplement: Supplementary file 1 — Supplementary Information [file 41467_2023_42344_MOESM1_ESM.pdf]

Supplementary Information for:

Title: Liquid spherical shells are a non-equilibrium steady state of active droplets

Authors: Alexander M. Bergmann,[1] Jonathan Bauermann,[2,3] Giacomo Bartolucci,[2,3] Carsten Donau,[1] Michele Stasi,[1] Anna-Lena Holtmannspötter,[1] Frank Jülicher, [2,3] Christoph A. Weber,[4,*] Job Boekhoven[1,*]

Affiliations:
[1]School of Natural Sciences, Department of Chemistry, Technical University of Munich, Lichtenbergstrasse 4, 85748 Garching, Germany.

[2]Max Planck Institute for the Physics of Complex Systems, Nöthnitzer Strasse 38, 01187 Dresden, Germany.

[3]Center for Systems Biology Dresden, Pfotenhauerstrasse 108, 01307 Dresden, Germany.

[4]Faculty of Mathematics, Natural Sciences, and Materials Engineering: Institute of Physics, University of Augsburg, Universitätsstrasse 1, 86159 Augsburg, Germany.

These authors contributed equally: Alexander M. Bergmann, Jonathan Bauermann, Giacomo Bartolucci, Carsten Donau

*corresponding author

Table of Contents

I. Supplementary Methods

- (A) Kinetic model
- (B) Thermodynamic model for the experimental phase diagram
- (C) Sharp interface model for the kinetics of active droplets and active spherical shells
- (D) Parameter values used in numerical calculations
- (E) Calculations of free energies and free energy rates

II. Supplementary Discussion

III. Supplementary Tables

IV. Supplementary Figures

V. Supplementary References

I. Supplementary Methods

(A) Kinetic model.

The reaction cycle in a homogeneous system is described in a kinetic model according to the following mechanism:

$$F \xrightarrow{k_0} W$$

$$Ac + F \xrightarrow{k_1} I$$

$$I \xrightarrow{k_{An}} An + W$$

$$I \xrightarrow{k_{Ac}} Ac + W$$

$$An \xrightarrow{k_2} Ac$$

Ac is the dicarboxylic acid, F is the fuel, I is the intermediate O-acylurea, W is the waste, and An is the anhydride.

The mechanism translates into the following set of differential equations:

$$\begin{cases} \frac{d[Ac]}{dt} = -k_1 \cdot [Ac] \cdot [F] + k_2 \cdot [An] + k_{Ac} \cdot [I] \\ \frac{d[F]}{dt} = -k_1 \cdot [Ac] \cdot [F] - k_0 \cdot [F] \\ \frac{d[W]}{dT} = +k_0 \cdot [F] + k_{Ac} \cdot [I] + k_{An} \cdot [I] \\ \frac{d[An]}{dt} = +k_{An} \cdot [I] - k_2 \cdot [An] \\ \frac{d[I]}{dt} = +k_1 \cdot [Ac] \cdot [F] - k_{An} \cdot [I] - k_{Ac} \cdot [I] \end{cases} \quad (1)$$

We then applied steady-state approximation to obtain:

$$\frac{d[I]}{dt} = +k_1 \cdot [Ac] \cdot [F] - k_{An} \cdot [I] - k_{Ac} \cdot [I] \approx 0 \Rightarrow [I] \approx \frac{k_1 \cdot [Ac] \cdot [F]}{k_{An} \cdot (\frac{k_{Ac}}{k_{An}} + 1)} \quad (2)$$

We called $\frac{k_{Ac}}{k_{An}} = K$ and used the relation above in the set of differential equations to obtain:

$$\begin{cases} \frac{d[Ac]}{dt} = -k_1 \cdot [Ac] \cdot [F] + k_2 \cdot [An] + \frac{K \cdot k_1 \cdot [Ac] \cdot [F]}{(K+1)} \\ \frac{d[F]}{dt} = -k_1 \cdot [Ac] \cdot [F] - k_0 \cdot [F] \\ \frac{d[W]}{dT} = +k_0 \cdot [F] + k_1 \cdot [Ac] \cdot [F] \\ \frac{d[An]}{dt} = +\frac{k_1 \cdot [Ac] \cdot [F]}{(K+1)} - k_2 \cdot [An] \end{cases} \quad (3)$$

Experimental data were fit to the equation system (2) using a custom program in Python 3 (kinmodel, https://github.com/scotthartley/kinmodel) previously published by the group of

Hartley and applied to similar systems[1]. To calculate concentrations of precursor and product under continuous fueling, the change in fuel concentration was set to 0 ($\frac{d[F]}{dt}$=0).

(B) Thermodynamic model for the experimental phase diagram

In the theoretical model, we use an effective ternary mixture where the influence of fuel, waste, and polyanion is accounted for in an implicit manner; details see section (C). The effective components of the ternary mixture are solvent, the precursor A, and the product B. For these components, equilibrium concentrations inside and outside were determined experimentally; see Supplementary Table 5 and 6. To fit the corresponding experimental diagram, we use a Flory-Huggins free energy density given as [2,3]

$$f(c_A, c_B) = k_B T \left[\sum_{i=A,B,S} c_i \log(r_i c_i) + \sum_{ij=AB,AS,BS} \chi_{ij}\, r_i c_i\, r_j c_j \right] , \tag{4}$$

where c_A and c_B denote the concentrations of components A and B, respectively. In Eq. (4), we have combined the molecular volumes ν_A, ν_B, and ν_S in the ratios introduced the molecular volume ratios $r_i = \nu_i/\nu_S$. The concentration of the solvent follows from the incompressibility of the mixture, $c_S = 1/\nu_S - r_A c_A - r_B c_B$. This free energy density depends on five parameters: the molecular volume rations, r_A and r_B, and the interaction parameters, χ_{AB}, χ_{AS}, and χ_{AB}. We determine these five parameters by fitting experimental measurements of different phase equilibria. At phase equilibria, the chemical potentials $\mu_i = \partial f/\partial c_i$ of components A and B and the osmotic pressure $\Pi = -f + \sum_{i=A,B} c_i \mu_i$ are balanced between the phases. For the measurements, the product was stabilised against hydrolysis by mutating the C terminal aspartic acid for an asparagine. This chemical modification yields a peptide that has the same interaction propensities as the product but is stable, *i.e.*, it does not convert to the precursor. Every measured point in the phase diagram gives three constraints

$$\mu_A\left(c_A^{\mathrm{I}}, c_B^{\mathrm{I}}\right) = \mu_A\left(c_A^{\mathrm{II}}, c_B^{\mathrm{II}}\right) , \tag{5}$$

$$\mu_B\left(c_A^{\mathrm{I}}, c_B^{\mathrm{I}}\right) = \mu_B\left(c_A^{\mathrm{II}}, c_B^{\mathrm{II}}\right) , \tag{6}$$

$$\Pi\left(c_A^{\mathrm{I}}, c_B^{\mathrm{I}}\right) = \Pi\left(c_A^{\mathrm{II}}, c_B^{\mathrm{II}}\right) . \tag{7}$$

We obtained the five unknown parameters by simultaneously minimizing the deviations for the

resulting 13 conditions. The best fit was obtained for $r_A = 35.1$, $r_B = 19.4$, $\chi_{AB} = -0.18$, $\chi_{AS} = 0.78$, and $\chi_{AS} = 1.29$. In Supplementary Fig. 9, we show the corresponding phase diagram together with the experimental measured concentration values. The last thermodynamic parameter needed for our model is the surface tension γ. Since γ is difficult to estimate within our experimental setup, we use the value $\gamma = 75\,\mu$N m^{-1} which is thousand times smaller than the air-water interfacial tension. Our value is in good agreement with surface tensions measured for similar coacervates.[4] Note that the used value is slightly larger than for biological condensates.[5]

(C) Sharp interface model for the kinetics of active droplets and active spherical shells

In general, diffusion is driven by spatial gradients of chemical potentials, while reactions minimize the difference in chemical potentials between products and reactants. For the following, we consider linearized kinetic equations, where diffusion is driven by spatial gradients of concentrations and the reaction

$$A \rightleftharpoons B\,, \tag{8}$$

is driven by the differences in concentrations of the respective components. The resulting kinetic parameters in terms of diffusivities and rate coefficients are then determined via experimental measurements, see experimental section. Therefore, the dynamical equation for c_A and c_B read [6,7]

$$\partial_t c_i^\alpha = D_i{}^\alpha \nabla^2 c_i{}^\alpha + k_i^\alpha c_j{}^\alpha - k_{ji}^\alpha c_i{}^\alpha\,. \tag{9}$$

In the case of spherical active droplets, there are two different domains with $\alpha =$ I,II, where I denotes the dense phase of droplet and II denotes the dilute phase outside the droplet. For spherical symmetric active spherical shells, three different domains exist, $\alpha =$ I,II,III, where I denotes the dilute phase within the core of the spherical shell, III labels the dense phase of the spherical shell, and II correspond to the dilute phase outside the spherical shell.

(C1) Chemically active droplet

We calculate the radial symmetric stationary profiles that solve Eq. (9) in the two different domains as a function of the radial coordinate r. At the droplet's core ($r = 0$), we impose no flux boundary conditions for the solution of domain I. Similarly, no flux boundary conditions are imposed at system boundary $r = R_{sys}$ for the solution in domain II. The two domains are coupled at the sharp interface at position $r = R$. Here, we impose concentration boundary conditions $c_A^\alpha(R) = a^\alpha$, $c_B^\alpha(R) = b^\alpha$. These concentrations are later determined self-consistently. For these boundary conditions, the stationary solutions for domain I read

$$c_A^{\mathrm{I}}(r) = \frac{b^{\mathrm{I}} - a^{\mathrm{I}}\rho_k^{\mathrm{I}}}{\rho_D^{\mathrm{I}} - \rho_k^{\mathrm{I}}} \frac{R \sinh\left(r/\lambda^{\mathrm{I}}\right)}{r \sinh\left(R/\lambda^{\mathrm{I}}\right)} + \frac{a^{\mathrm{I}}\rho_D^{\mathrm{I}} - b^{\mathrm{I}}}{\rho_D^{\mathrm{I}} - \rho_k^{\mathrm{I}}}, \tag{10}$$

$$c_B^{\mathrm{I}}(r) = \rho_D^{\mathrm{I}} \frac{b^{\mathrm{I}} - a^{\mathrm{I}}\rho_k}{\rho_D^{\mathrm{I}} - \rho_k^{\mathrm{I}}} \frac{R \sinh\left(r/\lambda^{\mathrm{I}}\right)}{r \sinh\left(R/\lambda^{\mathrm{I}}\right)} + \rho_k^{\mathrm{I}} \frac{a^{\mathrm{I}}\rho_D^{\mathrm{I}} - b^{\mathrm{I}}}{\rho_D^{\mathrm{I}} - \rho_k^{\mathrm{I}}}, \tag{11}$$

while in domain II, the stationary solutions follow

$$c_A^{\mathrm{II}}(r) = \frac{a^{\mathrm{II}}k_{BA}^{\mathrm{II}} - b^{\mathrm{II}}k_{AB}}{k_{BA}^{\mathrm{II}} - k_{AB}^{\mathrm{II}}\rho_D^{\mathrm{II}}} \, \frac{R}{r} \, \frac{\sinh\left(r/\lambda^{\mathrm{II}}\right) + \Phi \cosh\left(r/\lambda^{\mathrm{II}}\right)}{\sinh\left(R/\lambda^{\mathrm{II}}\right) + \Phi \cosh\left(R/\lambda^{\mathrm{II}}\right)} - \frac{a^{\mathrm{II}}k_{BA}^{\mathrm{II}} - b^{\mathrm{II}}k_{AB}\rho_D^{\mathrm{II}}}{k_{BA}^{\mathrm{II}} - k_{AB}^{\mathrm{II}}\rho_D^{\mathrm{II}}}, \tag{12}$$

$$c_B^{\mathrm{II}}(r) = \rho_D^{\mathrm{II}} \frac{a^{\mathrm{II}}k_{BA}^{\mathrm{II}} - b^{\mathrm{II}}k_{AB}}{k_{BA}^{\mathrm{II}} - k_{AB}^{\mathrm{II}}\rho_D^{\mathrm{II}}} \, \frac{R}{r} \, \frac{\sinh\left(r/\lambda^{\mathrm{II}}\right) + \Phi \cosh\left(r/\lambda^{\mathrm{II}}\right)}{\sinh\left(R/\lambda^{\mathrm{II}}\right) + \Phi \cosh\left(R/\lambda^{\mathrm{II}}\right)} - \rho_k^{\mathrm{II}} \frac{a^{\mathrm{II}}k_{BA}^{\mathrm{II}} - b^{\mathrm{II}}k_{AB}\rho_D^{\mathrm{II}}}{k_{BA}^{\mathrm{II}} - k_{AB}^{\mathrm{II}}\rho_D^{\mathrm{II}}}. \tag{13}$$

We have made use of $\lambda^\alpha = \sqrt{D_A^\alpha D_B^\alpha/(D_A^\alpha k_{AB}^\alpha + D_B^\alpha k_{BA}^\alpha)}$, $\rho_D^\alpha = -D_A^\alpha/D_B^\alpha$, and $\rho_k^\alpha = k_{BA}^\alpha/k_{AB}^\alpha$, where $\alpha = \mathrm{I}, \mathrm{II}$ indicate the phases.
The coefficient $\Phi = -(\lambda^{\mathrm{II}} R_{\mathrm{sys}} \cosh[\lambda^{\mathrm{II}} R_{\mathrm{sys}}] - \sinh[\lambda^{\mathrm{II}} R_{\mathrm{sys}}])/(\lambda^{\mathrm{II}} R_{\mathrm{sys}} \sinh[\lambda^{\mathrm{II}} R_{\mathrm{sys}}] - \cosh[\lambda^{\mathrm{II}} R_{\mathrm{sys}}])$ ensures the zero flux boundary condition at the system radius.

Finally, we have to determine the four interface concentrations a^{I}, a^{II}, b^{I}, b^{II} and the position of the interface R. For this, we need five constraints. Three of these constraints follow from the

assumption of a local equilibrium of phase separation [7]

$$\mu_A(a^{\mathrm{I}}, b^{\mathrm{I}}) = \mu_A(a^{\mathrm{II}}, b^{\mathrm{II}}), \tag{14}$$

$$\mu_B(a^{\mathrm{I}}, b^{\mathrm{I}}) = \mu_B(a^{\mathrm{II}}, b^{\mathrm{II}}), \tag{15}$$

$$\Pi(a^{\mathrm{I}}, b^{\mathrm{I}}) = \Pi(a^{\mathrm{II}}, b^{\mathrm{II}}) - \frac{2\gamma}{R}\,. \tag{16}$$

Furthermore, the total of A and B have to be conserved in the system,

$$V_{\mathrm{sys}}\,\bar{c}_{\mathrm{tot}} = \frac{4}{3}\pi\left[(a^{\mathrm{I}} + b^{\mathrm{I}})R^3 + (a^{\mathrm{II}} + b^{\mathrm{II}})(R^3_{\mathrm{sys}} - R^3)\right]\,, \tag{17}$$

where $c_{\mathrm{tot}} = c_A + c_B$ is the conserved quantity associated to the chemical reaction in Eq. (8). Finally, the conservation law at the interface in the stationary state requires

$$j_i^{\mathrm{I}}(R) = j_i^{\mathrm{II}}(R)\,, \tag{18}$$

where $j_i^\alpha = -D_i^\alpha \partial_r c_i^\alpha$ is radial component of the material flux density. Note that the stationary solution implies that $j_A^\alpha(r) = -j_B^\alpha(r)$. Thus, the constraint Eq. (18) is fulfilled simultaneously for both components A and B, making the total number of independent constraints equal to five.

(C2) Chemically active spherical shell

In the spherical shell state, the stationary concentration profiles in domain I and II have the same functional form as in the active droplet state. In addition, there is the shell domain III between domain I and domain II. Here, the stationary solutions of Eq. (9), with concentration boundary conditions on the interfaces left and right

$$c_A^{\mathrm{III}}(r) = K_1\frac{\sinh\left(r/\lambda^{\mathrm{III}}\right)}{r} + K_2\frac{\cosh\left(r/\lambda^{\mathrm{III}}\right)}{r} + K_3 + \frac{K_4}{r}, \tag{19}$$

$$c_B^{\mathrm{III}}(r) = \rho_D^{\mathrm{III}}\left[K_1\frac{\sinh\left(r/\lambda^{\mathrm{III}}\right)}{r} + K_2\frac{\cosh\left(r/\lambda^{\mathrm{III}}\right)}{r}\right] + \rho_k^{\mathrm{III}}\left[K_3 + \frac{K_4}{r}\right]\,. \tag{20}$$

Here, we have abbreviated

$$
\begin{aligned}
K_1 = \frac{\mathrm{csch}[(R_{\mathrm{out}} - R_{\mathrm{in}})/\lambda^{\mathrm{III}}]}{K^{\mathrm{III}}_{BA} - \rho^{\mathrm{III}}_D K^{\mathrm{III}}_{AB}} &\Big[(b^{\mathrm{III,in}} K^{\mathrm{III}}_{AB} - a^{\mathrm{III,in}} K^{\mathrm{III}}_{BA}) R_{\mathrm{in}} \cosh\Big(R_{\mathrm{out}}/\lambda^{\mathrm{III}}\Big) \\
&- (b^{\mathrm{III,out}} K^{\mathrm{III}}_{AB} - a^{\mathrm{III,out}} K^{\mathrm{III}}_{BA}) R_{\mathrm{out}} \cosh\Big(R_{\mathrm{in}}/\lambda^{\mathrm{III}}\Big)\Big] \,,
\end{aligned}
\tag{21}
$$

$$
\begin{aligned}
K_2 = \frac{\mathrm{csch}[(R_{\mathrm{out}} - R_{\mathrm{in}})/\lambda^{\mathrm{III}}]}{K^{\mathrm{III}}_{BA} - \rho^{\mathrm{III}}_D K^{\mathrm{III}}_{AB}} &\Big[(b^{\mathrm{III,out}} K^{\mathrm{III}}_{AB} - a^{\mathrm{III,out}} K^{\mathrm{III}}_{BA}) R_{\mathrm{out}} \sinh\Big(R_{\mathrm{in}}/\lambda^{\mathrm{III}}\Big)] \\
&- (b^{\mathrm{III,in}} K^{\mathrm{III}}_{AB} - a^{\mathrm{III,in}} K^{\mathrm{III}}_{BA}) R_{\mathrm{in}} \sinh\Big(R_{\mathrm{out}}/\lambda^{\mathrm{III}}\Big)\Big] \,,
\end{aligned}
\tag{22}
$$

$$
K_3 = \frac{(a^{\mathrm{III,out}} D^{\mathrm{III}}_A + b^{\mathrm{III,out}} D^{\mathrm{III}}_B) R_{\mathrm{out}} - (a^{\mathrm{III,in}} D^{\mathrm{III}}_A + b^{\mathrm{III,in}} D^{\mathrm{III}}_B) R_{\mathrm{in}})}{(D^{\mathrm{III}}_A + D^{\mathrm{III}}_B \rho^{\mathrm{III}}_k)(R_{\mathrm{out}} - R_{\mathrm{in}})} \,, \tag{23}
$$

$$
K_4 = \frac{(a^{\mathrm{III,in}} - a^{\mathrm{III,out}}) D^{\mathrm{III}}_A + (b^{\mathrm{III,in}} - b^{\mathrm{III,out}}) D^{\mathrm{III}}_B}{(D^{\mathrm{III}}_A + D^{\mathrm{III}}_B \rho^{\mathrm{III}}_k)(R_{\mathrm{out}} - R_{\mathrm{in}})} \, R_{\mathrm{in}} R_{\mathrm{out}} \,. \tag{24}
$$

We introduced $\lambda^{\mathrm{III}} = \sqrt{D^{\mathrm{III}}_A D^{\mathrm{III}}_B / (D^{\mathrm{III}}_A k^{\mathrm{III}}_{AB} + D^{\mathrm{III}}_B k^{\mathrm{III}}_{BA})}$, $\rho^{\mathrm{III}}_D = -D^{\mathrm{III}}_A / D^{\mathrm{III}}_B$, and $\rho^{\mathrm{III}}_k = k^{\mathrm{III}}_{BA} / k^{\mathrm{III}}_{AB}$, in analogy to phases I and II.

Finally, we have to determine the eight interface concentrations a^{I}, $a^{\mathrm{III,in}}$,$a^{\mathrm{III,out}}$, a^{II}, b^{I}, $b^{\mathrm{III,in}}$,$b^{\mathrm{III,out}}$, and b^{II}, and the two positions of the interfaces R_{in} and R_{out}. For this, we need ten constraints, three of which follow from the assumption of a local equilibrium of phase separation at R_{in}

$$
\mu_A(a^{\mathrm{I}}, b^{\mathrm{I}}) = \mu_A(a^{\mathrm{III,in}}, b^{\mathrm{III,in}}) \,, \tag{25}
$$

$$
\mu_B(a^{\mathrm{I}}, b^{\mathrm{I}}) = \mu_B(a^{\mathrm{III,in}}, b^{\mathrm{III,in}}) \,, \tag{26}
$$

$$
\Pi(a^{\mathrm{I}}, b^{\mathrm{I}}) = \Pi(a^{\mathrm{III,in}}, b^{\mathrm{III,in}}) - \frac{2\gamma}{R_{\mathrm{in}}} \,, \tag{27}
$$

and three from local equilibrium of phase separation at R_{out}

$$
\mu_A(a^{\mathrm{III,out}}, b^{\mathrm{III,out}}) = \mu_A(a^{\mathrm{II}}, b^{\mathrm{II}}) \,, \tag{28}
$$

$$
\mu_B(a^{\mathrm{III,out}}, b^{\mathrm{III,out}}) = \mu_B(a^{\mathrm{II}}, b^{\mathrm{II}}) \,, \tag{29}
$$

$$
\Pi(a^{\mathrm{III,out}}, b^{\mathrm{III,out}}) = \Pi(a^{\mathrm{II}}, b^{\mathrm{II}}) - \frac{2\gamma}{R_{\mathrm{out}}} \,. \tag{30}
$$

These six equations must be supplemented with a global conservation law

$$\bar{c}_{\text{tot}}\, V_{\text{sys}} = \int dV\, (c_A + c_B)\,, \tag{31}$$

a local conservation law at R_{in},

$$j_i^{\text{I}}(R_{\text{in}}) = j_i^{\text{II}}(R_{\text{in}})\,, \tag{32}$$

and a local conservation law at R_{out},

$$j_i^{\text{II}}(R_{\text{out}}) = j_i^{\text{III}}(R_{\text{out}})\,. \tag{33}$$

In contrast to domains I and II, in domain III, $j_A \neq -j_B$, due to the $1/r$ term in the solution of the Laplace equation. Thus, the two flux equations have to be balanced for the components A and B at one interface, respectively. Due to the symmetry of the stationary solutions, one equality is automatically fulfilled at the second interface. Thus, we are left with three independent constraints coming from the local conservation laws at the interfaces Eqs. (32) and (33).

(D) Parameter values used in numerical calculations

If not indicated otherwise, we chose the parameters indicated in Supplementary Table 7 for the numerical calculations. For the figures shown in this work, we fixed the following parameter values as stated in Supplementary Table 8. To illustrate the dependency of the stationary profiles on the parameters, we show the concentration profiles in composition space for different settings of the ratio of $D_A^{\text{II}}/D_B^{\text{II}}$, the surface tension γ, and the ratio of the activation rates $k_{BA}^{\text{I}}/k_{BA}^{\text{II}}$ in Supplementary Fig. 12.

(E) Calculations of free energies and free energy rates

We can estimate the free energy difference between a spherical shell stationary state and the

corresponding homogeneous state with the same average concentrations

$$\bar{c}_i = \frac{1}{V_{\text{sys}}} \int dV \, c_i \, , \tag{34}$$

with $i = A, B$. In the following estimates, we will consider spherical shells at steady state corresponding to $R_{\text{sys}} = 35\,\mu$m. We start from the concentrations in the coexisting phases, c_i^+ and c_i^-, corresponding to the spherical shell average concentration $\bar{c}_i$. Neglecting the interface contribution and considering each phase homogeneous, the free energy in the spherical shell state can be estimated directly via

$$F_{\text{mix}} = V^+ f^+ + (V_{\text{sys}} - V^-) f^- \, , \tag{35}$$

where the free energy density in each phase is $f^\pm = f(c_A^\pm, c_B^\pm)$ and V^+ is the total volume of the dense phase. With the parameters displayed in Supplementary Table 7, this difference yields $F_{\text{mix}} = 2$ nJ.

This free energy can be compared with the total activation free energy, defined as the energy of a B molecule times the number of excess B molecules at the spherical shell steady state

$$F_{\text{act}} = \Delta\omega(\bar{c}_B - c_B^0) V_{\text{sys}} \, , \tag{36}$$

where $c_B^0 = c_{\text{tot}}/[1 + \exp(\Delta\Omega)]$ is the B concentration in the homogeneous equilibrium state, i.e., without fuel present, and $\Delta\Omega$ is the activation free energy of a single A molecule, which is roughly $10\,k_BT$ [8] As outlined in the main text, making use of the parameters in Supplementary Table 7, we estimate $F_{\text{act}} \simeq 80$ nJ.

Next, we estimate the number of particles that constantly get converted from the precursor A into the product B, per unit time and volume,

$$n_{\text{tot}} = \frac{1}{V_{\text{sys}}} \int dV \; c_A \, k_{BA} \, , \tag{37}$$

which determines the total free energy turned over per time and volume

$$J_{\text{tot}} = \Delta\Omega\, n_{\text{tot}}\,. \tag{38}$$

Here, $\Delta\Omega$ denotes the activation energy supplied by fuel to the precursor to form a product. Using the parameters shown in Supplementary Table 7, $c_{\text{F}} = 8.6$ mM, and $\Delta\Omega \simeq 10 k_B T$, we obtain $n_{\text{tot}} = 4 \cdot 10^6\,\text{s}^{-1}\mu\text{m}^{-1}$ and $J_{\text{tot}} = 0.25$ W/L as outlined in the main text.

We can also estimate the free energy flux through the spherical shell interface. Specifically, we calculate the flux of product B through the interface where each product is activated by $\Delta\Omega$

$$J_{\text{int}} = \left[4\pi R_{\text{out}}^2\, j_A^{\text{II}}(R_{\text{out}}) - 4\pi R_{\text{in}}^2\, j_A^{\text{I}}(R_{\text{in}})\right] \Delta\omega / V_{\text{sys}}\,. \tag{39}$$

Using the parameters shown in Supplementary Table 7, and $\Delta\omega \sim 10\, k_B T$, this leads to an energy influx per unit time of $J_{\text{int}} = 0.198$ W/L. Comparing it to the total power J_{tot}, this leads to a ratio $J_{\text{int}}/J_{\text{tot}}$ around 0.7. In Supplementary Fig. 13, we explored how J_{tot} and J_{int} vary upon changes in the activation rate, induced by changes in the fuel concentration. Note that in our minimal model, the fuel implicitly enters the activation rate. As expected, J_{tot}, which represents the total power needed to activate precursor A to product B to their respective values at the non-equilibrium steady state (see definition in Eq. (38)), varies linearly with the fuel concentration. On the other hand, the free energy transported to the spherical shell interface, J_{int}, scales sublinearly with fuel concentration. This is because the higher the fuel the more activation occurs inside, making the B particle flux at the interface less pronounced. As a consequence, the ratio $J_{\text{int}}/J_{\text{tot}}$ decreases as fuel concentration increases.

II. Supplementary Discussion

Supplementary Discussion 1. We quantified the interaction strength between the peptides (precursor and product) with pSS by means of ITC. We used Ac-F(RG)$_3$N-NH$_2$ (product*) as a measure for the product (anhydride of precursor) since both molecules are structurally very similar and contain the same positive net charge.[10] Furthermore, product* was also used as a substitute for the product in the formation of static droplets, *i.e.*, droplets whose formation is not governed by chemical reactions.

Supplementary Discussion 2. For the mechanism evaluation, we wanted to quantify the precursor, product, and fuel partitioning in the active droplets. However, for the setup of continuously fueled droplets, a centrifugation assay is not directly possible. Determining the partitioning of the molecules in droplets fueled with a batch of fuel is possible for a specific time point with a centrifugation assay. Still, the molecular composition of the dilute and the dense phase is time-dependent (with respect to fuel addition), and thus, this data cannot be used to build a phase diagram. Furthermore, a proceeding of the reactions cannot be prevented during the preparation of these assays. To avoid this time dependence, we prepared passive droplets containing only one of the reactive components at a time. For the precursor and the product, passive droplets at different ratios of precursor and product* (without fuel) were used to measure active droplets in a steady state. To determine the partitioning of the fuel, we prepared droplets consisting only of product* and pSS (no precursor), which were therefore passive as well.

Supplementary Discussion 3. To test the robustness of the spherical shell formation, we tested different types of poly anions, different chain lengths of pSS, different pSS and precursor concentrations, and the influence of the dye (Supplementary Tables 9-12). For the polyanion polyvinyl sulfonate (pVS), no spherical shell formation was observed. We hypothesize that this is the case because no droplets larger than $V_{unstable}$ were formed (Supplementary Fig. 14a). For the polyanion polyuridylic acid (pU), no spherical shells were observed either, even though droplets larger than $V_{unstable}$ were obtained (Supplementary Fig. 14b). Most likely, the previously observed meta-stability of pU-based droplets plays a significant role in the prevention of spherical shell formation.[11] Under the screened conditions, only pSS-based droplets grew sufficiently large while preserving their fuel-responsive nature to form spherical shells. However, we observed no evidence that other polyanions that yield coacervate-based droplets with similar properties to pSS-based droplets could not form spherical shells.

The reduction of the pSS chain length from 75 kg mol^{-1} to 32 kg mol^{-1} had no significant effect

on the spherical shell formation (Supplementary Fig. 14c). However, no spherical shells could be obtained upon increasing the chain length of pSS to 150 kg mol^{-1} due to the formation of droplets prior to the addition of DIC.

Similarly, a reduction in the pSS concentration led to the formation of droplets prior to the addition of DIC. For pSS concentrations of 5 and 7 mM, spherical shell formation was observed, showing no significant difference in the formation (Fig.3i and Supplementary Fig. 14f). For 10 mM pSS, only small droplets were formed that did not fuse into one droplet even after 5 h (Supplementary Fig. 14g), preventing proper analysis of spherical shell formation. Last, we used the labeled precursor NBD-G$(RG)_3$D-OH (Supplementary Fig. 14h), which showed no significant difference in the spherical shell formation in comparison to sulforhodamine B, verifying that the dye has no significant effect on the formation of the shells.

Supplementary Discussion 4. Upon decreasing the temperature, all droplets were stable, independent of their size, and we observed no spherical shell formation (Supplementary Fig. 15a). However, spherical shells that were already formed under standard conditions also appeared to be stable and did not revert to spherical droplets upon a decrease in temperature. This observation is coherent with the fact that non-equilibrium systems can settle in different steady states depending on the initial conditions, as can be seen in Fig.4g-i, but it could also be due to a metastable or kinetically trapped state of the shells. Due to the temperature dependence of the reaction rate constants, the diffusion, and the phase separation behavior of our system, no clear conclusion could be drawn about the effect of either of these parameters individually. Additionally, extensive wetting of the coacervate-based droplets prevented any reasonable analysis at elevated temperatures.

Similarly, the strong dependence of multiple parameters on the pH prevented a thorough investigation of its influence on the spherical shell formation. On the one hand, lowering the pH leads to the protonation of the precursor's carboxylic acids. This negation of negative charges is, like the anhydride formation, able to induce phase separation (Supplementary Fig. 15b). On the other hand, an increase in the pH prevented phase separation upon the addition of fuel which is most likely due to an insufficient product yield (Supplementary Fig. 15b).

III. Supplementary tables

reaction constant	Value
k_0	$2.9 \cdot 10^{-4}$ s^{-1}
k_1	$1.7 \pm 0.08 \cdot 10^{-1}$ M^{-1} s^{-1}
k	1.34 ± 1.0
k_2	$1.2 \pm 0.9 \cdot 10^{-2}$ s^{-1}

Supplementary Table 1: Kinetic parameters determined by the kinetic model.

	Precursor	Prodcut*
K_D (μM)	104.5 ± 11.4	2.4 ± 1.2
Δ*G* (kcal mol^{-1})	-5.4 ± 0.1	-7.7 ± 0.3

Supplementary Table 2: Experimentally determined dissociation constant K_D and free energy Δ*G* of the interaction between precursor, product*, and pSS.

Diffusivity	Spherical shell	Droplet
$D_{precursor}$ (μm^2 s^{-1})	0.060 ± 0.010	0.042 ± 0.003
$D_{product*}$ (μm^2 s^{-1})	0.029 ± 0.008	0.032 ± 0.003

Supplementary Table 3: Experimentally determined values for the diffusivities in the active droplet and the active spherical shell.

Total concentrations			Fuel			
Precursor (mM)	Product* (mM)	pSS (mM)	c_{in} (mM)	c_{out} (mM)	V_{coa}/V_{dilute} (vol%)	$K_{partitioning}$ (c_{in}/c_{out})
0	2	5	14.4 ± 17.3	9.98 (99.8%)	0.10 ± 0.01	1.44 ± 1.73

Supplementary Table 4: Experimentally determined values for the partitioning of the fuel in passive droplets.

Total concentrations			Product*			
Precursor (mM)	Product* (mM)	pSS (mM)	c_{in} (mM)	c_{out} (mM)	V_{coa}/V_{dilute} (vol%)	$K_{partitioning}$ (c_{in}/c_{out})
20	0	5	-	-	-	-
9.5	0.5	5	176 ± 48	0.11 ± 0.03 (23%)	0.22 ± 0.04	1545 ± 904
9	1	5	233 ± 35	0.07 ± 0.02 (7%)	0.40 ± 0.05	3360 ± 1645
8	2	5	692 ± 139	0.10 ± 0.0 (5%)	0.28 ± 0.05	7146 ± 3258
0	2	5	699 ± 434	0.78 ± 0.08 (39%)	0.18 ± 0.05	901 ± 653

Supplementary Table 5: Experimentally determined values for the partitioning of the product* in passive droplets for the phase diagram.

Total concentrations			Precursor			
Precursor (mM)	Product* (mM)	pSS (mM)	c_{in} (mM)	c_{out} (mM)	V_{coa}/V_{dilute} (vol%)	$K_{partitioning}$ (c_{in}/c_{out})
20	0	5	309 ± 323	18.2 ± 1.5 (91%)	0.57 ± 0.10	17 ± 19
9.5	0.5	5	782 ± 584	7.8 ± 0.9 (82%)	0.22 ± 0.04	101 ± 88
9	1	5	734 ± 229	6.1 ± 0.5 (67%)	0.40 ± 0.05	121 ± 49
8	2	5	655 ± 236	6.2 ± 0.3 (78%)	0.28 ± 0.05	106 ± 43
0	2	5	-	-	-	-

Supplementary Table 6: Experimentally determined values for the partitioning of the precursor in passive droplets for the phase diagram.

Quantity	Symbol	Value	Reference
Interaction parameter precursor - product	χ_{AB}	-0.18	Suppl. Methods (E
Interaction parameter precursor - solvent	χ_{AS}	0.78	Suppl. Methods (E
Interaction parameter product - solvent	χ_{BS}	1.28	Suppl. Methods (E
Precursor relative molecular volume	r_A	35.1	Suppl. Methods (E
Product relative molecular volume	r_B	19.4	Suppl. Methods (E
Surface tension	γ	75 µN m^{-1}	Suppl. Methods (E
Activation rate outside the drop	k^{II}_{BA}	0.17 c_{F} M^{-1}s^{-1}	Suppl. Table 1
Activation rate inside the drop	k^{I}_{BA}	0.51 c_{F} M^{-1}s^{-1}	Suppl. Table 1
Deactivation rate outside the drop	k^{II}_{AB}	0.012 s^{-1}	Suppl. Table 1
Deactivation rate inside the drop	k^{I}_{AB}	0.012 s^{-1}	Suppl. Table 1
Diffusion coefficient of A outside the drop	D^{II}_A	300 µm^2 s^{-1}	[9]
Diffusion coefficient of B outside the drop	D^{II}_B	300 µm^2 s^{-1}	[9]
Diffusion coefficient of A inside the drop	D^{I}_A	0.04 µm^2 s^{-1}	Suppl. Table 3
Diffusion coefficient of B inside the drop	D^{I}_B	0.0073 µm^2 s^{-1}	Suppl. Table 3
Activation rate outside the spherical shell	k^{II}_{BA}	0.17 c_{F} M^{-1}s^{-1}	Suppl. Table 1
Activation rate in the spherical shell shell	k^{III}_{BA}	0.51 c_{F} M^{-1}s^{-1}	Suppl. Table 1
Activation rate in the spherical shell core	k^{I}_{BA}	0.17 c_{F} M^{-1}s^{-1}	Suppl. Table 1
Deactivation rate outside the spherical shell	k^{II}_{AB}	0.012 s^{-1}	Suppl. Table 1
Deactivation rate in the spherical shell shell	k^{III}_{AB}	0.012 s^{-1}	Suppl. Table 1
Deactivation rate in the spherical shell core	k^{I}_{AB}	0.012 s^{-1}	Suppl. Table 1
Diffusion coefficient of A outside the spherical shell	D^{II}_A	300 µm^2 s^{-1}	[9]
Diffusion coefficient of B outside the spherical shell	D^{II}_B	300 µm^2 s^{-1}	[9]
Diffusion coefficient of A in the spherical shell shell	D^{III}_A	0.04 µm^2 s^{-1}	Suppl. Table 3
Diffusion coefficient of B inside spherical shell shell	D^{III}_B	0.0073 µm^2 s^{-1}	Suppl. Table 3
iffusion coefficient of A inside the spherical shell core	D^{I}_A	300 µm^2 s^{-1}	[9]
iffusion coefficient of B inside the spherical shell core	D^{I}_B	300 µm^2 s^{-1}	[9]

Supplementary Table 7: Table with input parameters used in the numerical calculations.

Figure	Quantity	Symbol	Value
Fig. 4 d and e	System radius	R_{sys}	35 μm
Fig. 4 d and e	Fuel concentration	c_{fuel}	8.6 mM
Fig. 4 f	System radius	R_{sys}	17.5 μm
Fig. 4 f	Fuel concentration	c_{fuel}	8.6 mM
Fig. 4 h	System radius	R_{sys}	25 μm
Fig. 4 h	Fuel concentration	c_{fuel}	10 mM
Fig. 4 i	System radius	R_{sys}	25 μm
Fig. 4 i	Fuel concentration	c_{fuel}	10 mM
Fig. 4 i	Dif. coef. of A in inside the shell	D_A^{III}	as indicated by $D_{\text{Precursor}}$
Fig. 4 i	Dif. coef. of B in inside the shell	D_B^{III}	D_A^{III}/5.5
Suppl. Fig. 10 a-c	System radius	R_{sys}	25 μm
Suppl. Fig. 10 b, c	Fuel concentration	c_{fuel}	8.6 mM
Suppl. Fig. 12 a-d	System radius	R_{sys}	20 μm
Suppl. Fig. 12 b	Ratio of dif. inside the drople	$\frac{D_A^{\text{II}}}{D_A^{\text{II}}}$	$\{1, 2, 4, 5.5, 10\}$ (ind. by arrow)
Suppl. Fig. 12 c	Surface tension	γ	$\{25\mu\text{N}, 75\mu\text{N}, 175\mu\text{N}\}$ (ind. by arrow)
Suppl. Fig. 12 d	Ratio of act. rates	$\frac{k_{\text{BA}}^{\text{I}}}{k_{\text{BA}}^{\text{II}}}$	$\{0.1, 3, 10\}$ (ind. by arrow)
Suppl. Fig. 13	System radius	R_{sys}	35 μm

Supplementary Table 8: Table with input parameters used for specific figures.

pAnion	pSS	pVS	pU
Observation	Shells	No shells	No shells

Supplementary Table 9: Observations upon changing the polyanion type. Standard conditions were 10 mM precursor, 5 mM polyanion, and 200 mM MES at pH 5.3 with 0.1 µM sulforhodamine B. 0.5 M DIC was added to the oil phase to achieve a steady state.

pSS length	32 kg mol^{-1}	75 kg mol^{-1}	150 kg mol^{-1}
Observation	Shells	Shells	Preassemblies

Supplementary Table 10: Observations upon changing the pSS chain length. Standard conditions were 10 mM precursor, 5 mM pSS, and 200 mM MES at pH 5.3 with 0.1 µM sulforhodamine B. 0.5 M DIC was added to the oil phase to achieve a steady state.

[pSS]	3 mM	5 mM	7 mM	10 mM
Observation	Preassemblies	Shells	Shells	No fusion

Supplementary Table 11: Observations upon changing the pSS concentration. Standard conditions were 10 mM precursor, x mM pSS, and 200 mM MES at pH 5.3 with 0.1 µM sulforhodamine B. 0.5 M DIC was added to the oil phase to achieve a steady state.

[Precursor]	8 mM	10 mM	12 mM	14 mM
Observation	Shells	Shells	Shells	Preassemblies

Supplementary Table 12: Observations upon changing the precursor concentration. Standard conditions were x mM precursor, 5 mM pSS, and 200 mM MES at pH 5.3 with 0.1 µM sulforhodamine B. 0.5 M DIC was added to the oil phase to achieve a steady state.

IV. Supplementary Figures

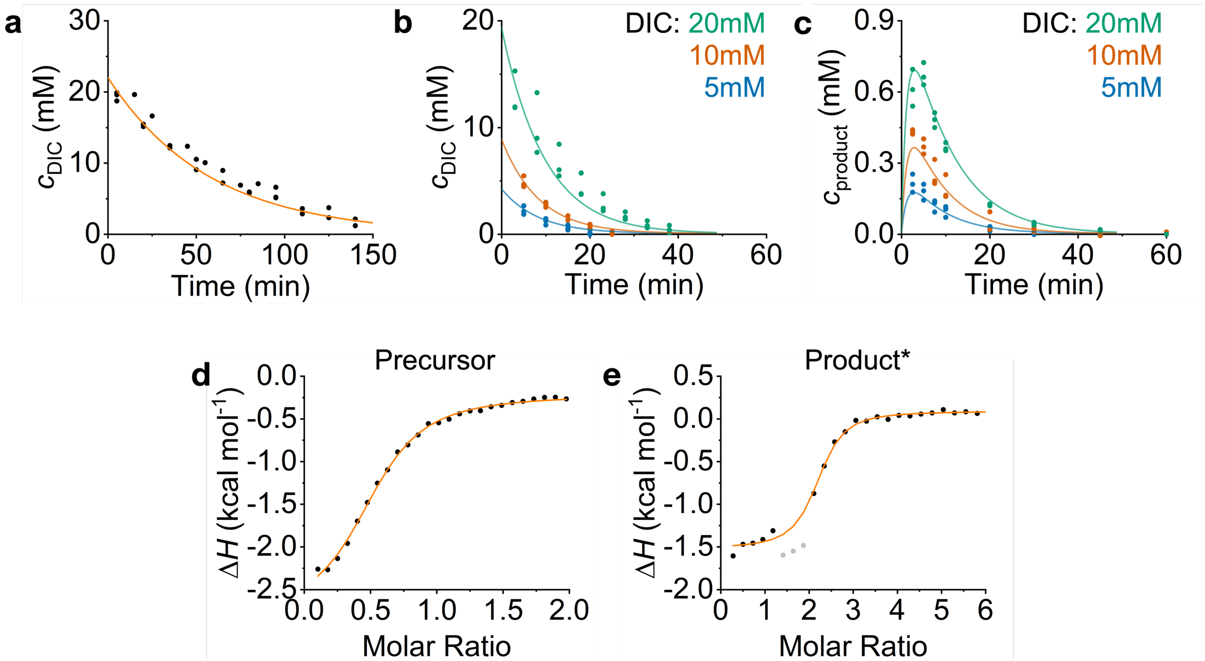

Supplementary Figure 1. Determination of the reaction constants and binding affinities. All experiments were performed in triplicate (*N*=3). **a.** Kinetics of 20 mM DIC hydration in 200 mM MES buffer at pH 5.3 measured by NMR. **b.** Kinetics of the DIC hydration catalyzed by the precursor. Conditions were 10 mM precursor and 200 mM MES at pH 5.3. Solid lines represent the fits of the kinetic model. **c.** Formation of the product upon addition of DIC measured by HPLC. Conditions were 10 mM precursor and 200 mM MES at pH 5.3. Solid lines represent the fits of the kinetic model. **d-e.** The change in enthalpy measured by ITC for the interaction between precursor (**d**) or product* (**e**) and pSS. Gray data points were not included in the fitting because they showed signs of coacervation. The solid lines represent the fit of the PEAQ-ITC Analysis software.

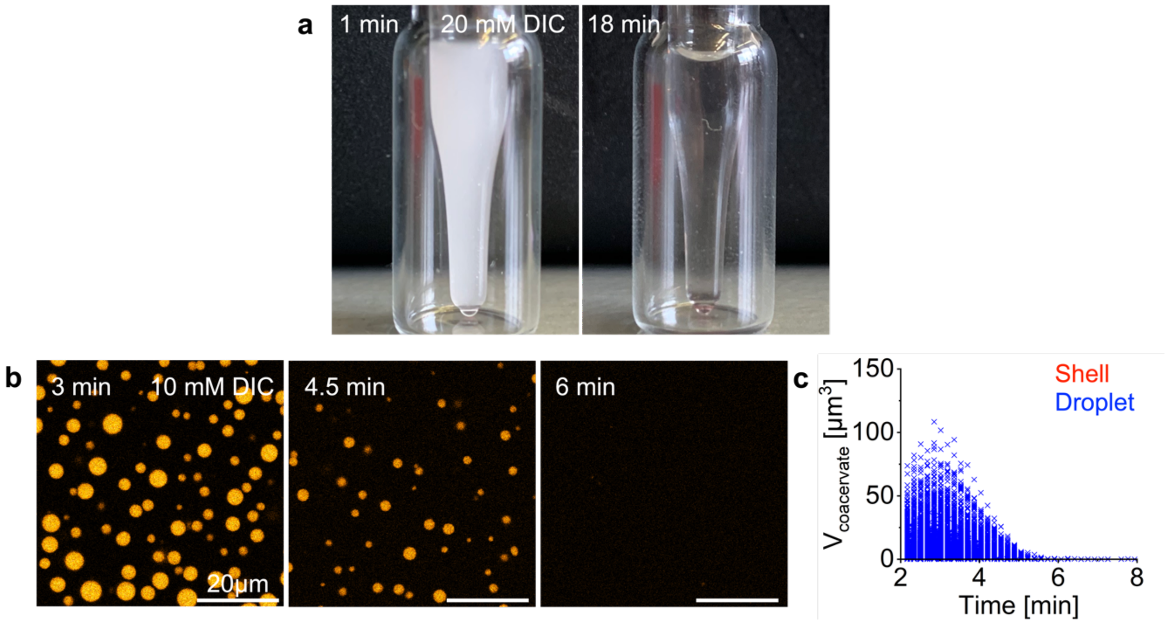

Supplementary Figure 2. Batch fueling of active droplets. a. Images of an HPLC inlet containing a solution of 22 mM precursor, 12 mM pSS, and 0.1 µM sulforhodamine B in 200 mM MES buffer at pH 5.3 fueled with 20 mM DIC. After adding the fuel, the sample turned turbid (1 min), and a clear solution was obtained again after 18 min. **b.** Confocal microscopy of a solution from A with 0.1 µM sulforhodamine B and fueled with 10 mM DIC. The active droplets grew and fused initially, but their size stayed below the size of the active droplets fueled with 20 mM DIC (Fig. 1b, c). After around 4 min, they started to dissolve without forming spherical shells. Imaging was done in PVA-coated ibidi chambers. All experiments were performed in triplicate (*N*=3). **c.** The volume of each active droplet (blue) is plotted for each time point. No spherical shells (red) were observed. Experiments were performed in triplicate (*N*=3).

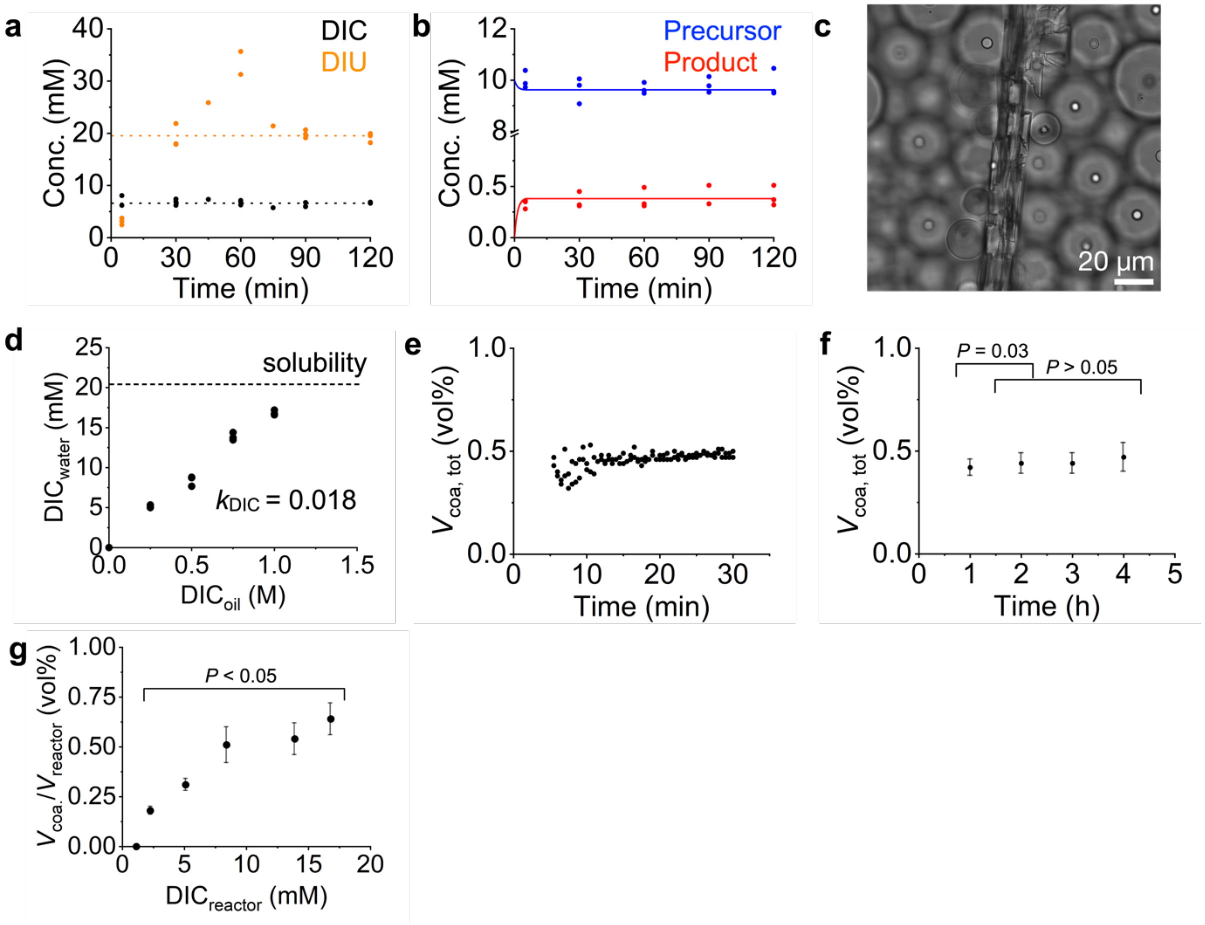

Supplementary Figure 3. Total coacervate volumes and reactant concentrations in a steady state. a. DIC (fuel) and DIU (waste) concentrations under steady-state conditions were determined by NMR. Conditions were 10 mM precursor and 200 mM MES at pH 5.3. To achieve a steady state, perfluorinated oil containing 0.5 M DIC was added to the aqueous phase. The dotted line represents the average DIC concentration of 6.6 mM. Measurements were performed in triplicate (*N*=3). **b.** Analytical HPLC determined average precursor and product concentrations under steady-state conditions. Conditions were 10 mM precursor and 200 mM MES at pH 5.3. To achieve a steady state, perfluorinated oil containing 0.5 M DIC was added to the aqueous phase. Solid lines represent the steady-state concentration of DIC calculated by the kinetic model of 6.6 mM. **c.** A representative bright field image of a DIU crystal formed in the oil phase during a steady-state experiment. **d.** The dependency of the DIC concentration in the oil phase on the DIC concentration in the aqueous phase. The aqueous phase consisted of 200 mM MES at pH 5.3, and the oil phase of Novec 7500 perfluorinated oil. The dotted line represents the DIC concentration in the aqueous phase if an excess of pure DIC was added on top of the aqueous phase without the oil phase. The average partitioning coefficient of DIC between the aqueous phase and the oil phase was k_{DIC} = 0.018. **e.** Total coacervate volume in one microfluidic reactor in the first 30 min after adding fuel. Conditions were 10 mM precursor, 5 mM pSS, and 200 mM MES at pH 5.3 with 0.5 M DIC. Data points are from two independent experiments. **f.** Average total coacervate volume of different microfluidic reactors over 4 h. Conditions were the same as in **e.** Error bars represent the standard deviation. *P*-values were calculated for α = 0.05 (n_{1h} = 41, n_{2h} = 41, n_{3h} = 35, n_{4h} = 49; $P_{2,3}$ = 0.69, $P_{3,4}$ = 0.07) **g.** The total volume of coacervate-based droplets within every microreactor is given dependent on the applied steady-state concentration of fuel. Microreactors were imaged 3 h after the addition of fuel. Error bars represent the standard. *P*-values were calculated for α = 0.05 ($n_{2.3\ mM}$ = 13,$n_{5.1\ mM}$ = 119, $n_{8.4\ mM}$ = 91, $n_{13.9\ mM}$ = 77, $n_{16.8\ mM}$ = 98; $P_{2.3,\ 5.1}$ = 0.00, $P_{5.1,\ 8.4}$ = 0.00, $P_{8.4,\ 13.9}$ = 0.02, $P_{13.9,\ 16.8}$ = 0.00).

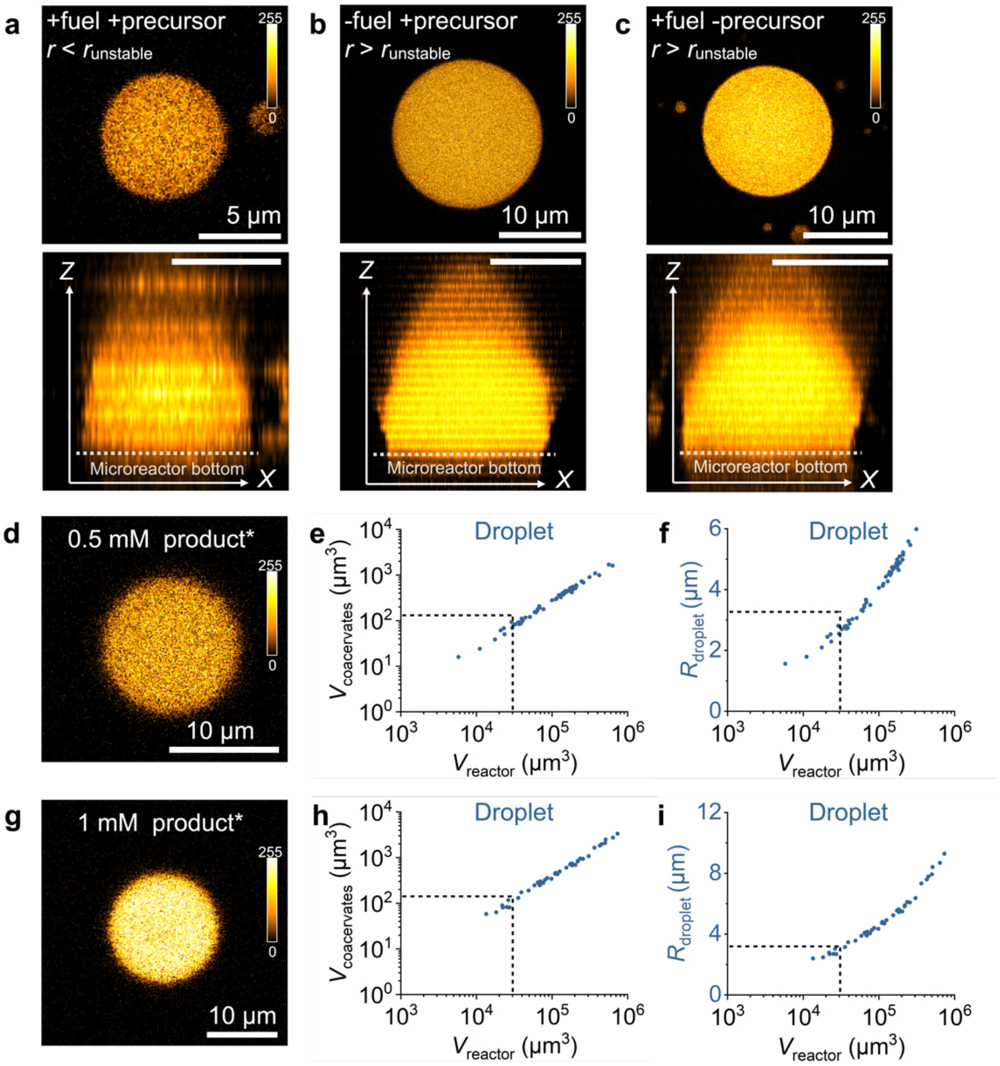

Supplementary Figure 4. Wetting of active and passive droplets without transitioning into a shell. All experiments were performed in microfluidic reactors. 5 mM pSS, 200 mM MES at pH 5.3, and 0.1 μM sulforhodamine B as a dye were used. All experiments were performed in triplicate (*N*=3). **a-c.** Representative images show the *XY*-plane at the center of the droplet to demonstrate that no spherical shell was formed and the *XZ*-projection of the droplet to demonstrate the wetting at the bottom of the microreactor. **a.** A representative active droplet with $r < r_{unstable}$ is shown 6 h after adding fuel. In addition to the abovementioned conditions, the sample contained 10 mM precursor, and 0.5 M DIC was added to the oil phase to achieve a steady state. **b.** A representative passive droplet with $r > r_{unstable}$ is shown 6 h after the induction of droplet formation. Conditions were 9 mM precursor, 1 mM product* with no DIC in the oil phase. Additionally, 1 w% Krytox 157 FSH was added to the oil phase. **c.** A representative passive droplet with $r > r_{shell}$ is shown 6 days after the induction of droplet formation. These passive droplets consist only of product* and pSS without the precursor. Since the added fuel cannot react with the product*, these droplets are thus passive. Conditions were 0 mM precursor, 2 mM product* with 0.5 M DIC in the oil phase to achieve a steady state. **d-i.** Images were acquired 4 h after the induction of droplet formation. Dashed lines represent the container size, coacervate volume, and size upon which spherical shell formation was observed for active droplets (Fig. 3i and j). **d-f.** Conditions were 9.5 mM precursor and 0.5 mM product*. **g-i.** Conditions were 9 mM precursor and 1 mM product*. To show that passive droplets do not transition into shells, a micrograph of a representative passive coacervate-based droplet (**d, g**), the volume of the total coacervate material (**e, h**) for every individual microreactor, and the radius (**f, i**) of the passive droplet in every individual microreactor is shown.

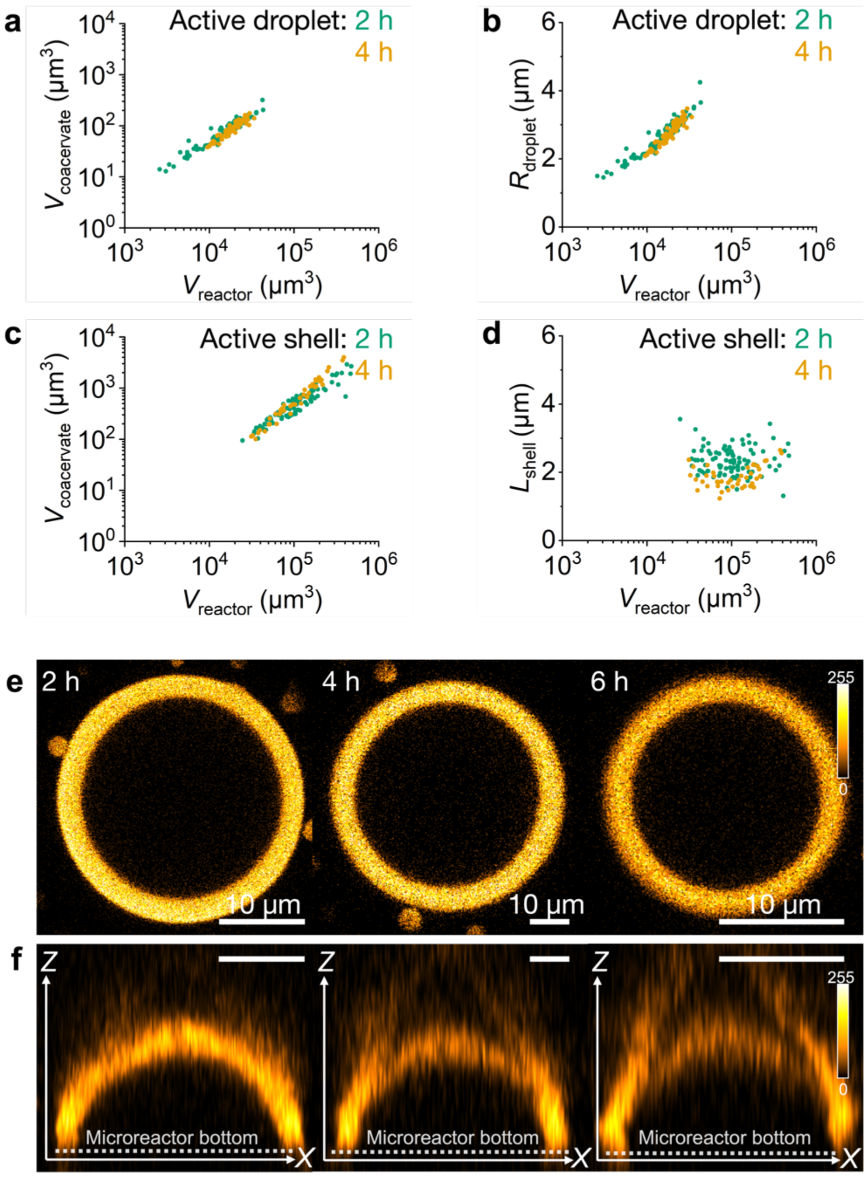

Supplementary Figure 5. Stability of active spherical shells. All experiments were performed in microreactors. Conditions were 10 mM precursor, 5 mM pSS, and 200 mM MES at pH 5.3 with 0.1 µM sulforhodamine B. 0.5 M DIC was added to the oil phase to achieve a steady state. All experiments were performed in triplicate (*N*=3). **a, c.** The volume of the total coacervate material 2 h and 4 h after the induction of coacervation is shown for every individual microreactor that contained an active droplet (**a**) or an active shell (**c**). The volume of active droplets and active shells, as well as the threshold upon which shells were formed, did not change between 2 h and 4 h. **b, d.** The radius of the active droplet (**b**) as well as the shell thickness L_{shell} of the active shell (**d**) in every individual microreactor is shown 2 h and 4 h after the induction of coacervation. The radius of active droplets and L_{shell} of active shells, and the threshold upon which shells are formed did not change between 2 h and 4 h. **e-f.** A representative active shell 2 h, 4 h, and 6 h after the induction of coacervation. In **e** the *XY*-plane of an active shell is shown, and in **f** the *XZ*-plane through the middle of the 3D projection of the respective active shell is shown. The scale bar of all images represents 10 µm. The color scale is given next to the images.

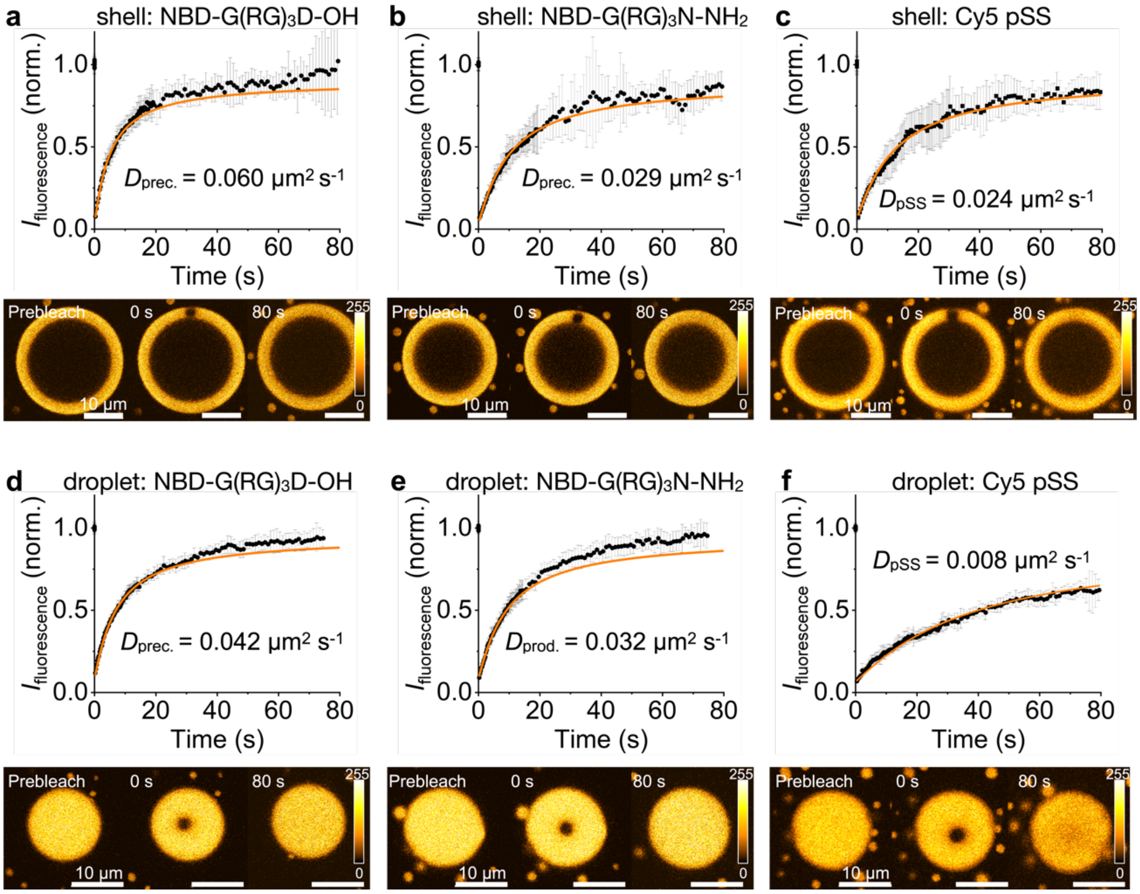

Supplementary Figure 6. FRAP of active droplets and active spherical shells. All experiments were performed in microreactors. Conditions were 10 mM precursor, 5 mM pSS, and 200 mM MES at pH 5.3 with 1 µM of the respective dye. 0.5 M DIC was added to the oil phase to achieve a steady state. Error bars represent the standard deviation of 10 different measurements (*N*=10). The time series shows the fluorescence recovery of a representative active shell. **a-c.** FRAP of the labeled precursor (**a**), labeled product* (**b**), and labeled pSS (**c**) in shells 3 h after the induction of coacervation. Representative images are shown for every measurement. The scale bar of all images represents 10 µm. The color scale is given next to the images. **d-f.** FRAP of the labeled precursor (**d**), the labeled product* (**e**), and of the labeled pSS (**f**) in active droplets 1 h after the induction of coacervation. Active droplets were imaged before they transitioned into a spherical shell. Representative images are shown for every measurement. The scale bar of all images represents 10 µm. The color scale is given next to the images.

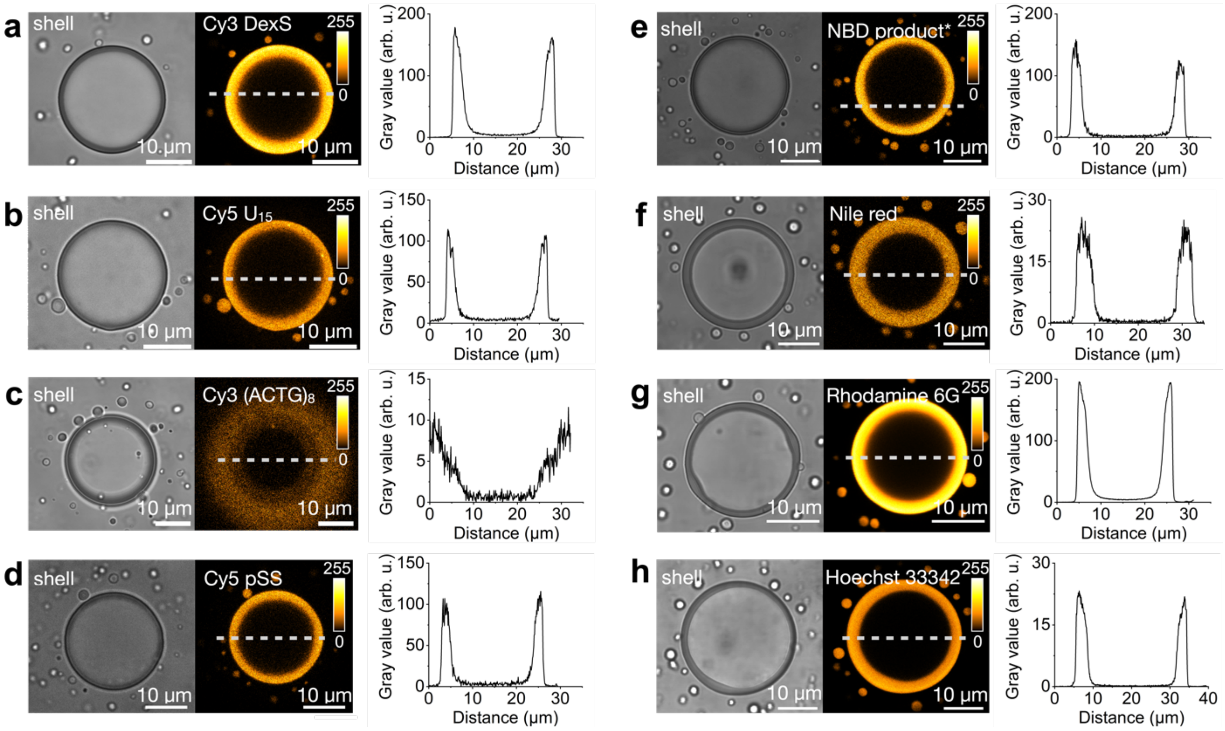

Supplementary Figure 7. Partitioning of fluorescent molecules into the active spherical shells. Conditions were 10 mM precursor, 5 mM pSS, and 200 mM MES at pH 5.3 with 1 µM of the respective dye. 0.5 M DIC was added to the oil phase to achieve a steady state. Active spherical shells were imaged 3 h after the induction of coacervation. The color scale is given next to the images. **a-h.** Bright-field image of an active shell containing Cy3 labeled dextran sulfate (Cy3 DexS, excitation at 552 nm, **a**), Cy5 labeled 15-mer of oligouridylic acid (Cy5 U_{15}, excitation at 638 nm, **b**) Cy3 labeled $(ATCG)_8$ (Cy3 $(ATCG)_8$, excitation at 552 nm, **c**), Cy5 labeled pSS (Cy5 pSS, excitation at 638 nm, **d)**, NBD labeled product* (excitation at 488 nm, **e**). The line profile of the fluorescence along the dotted line is shown, Nile red (excitation at 561 nm, **f**), rhodamine 6G (excitation at 488 nm, **g**), and Hoechst 33342 (excitation 561 nm, **h**).

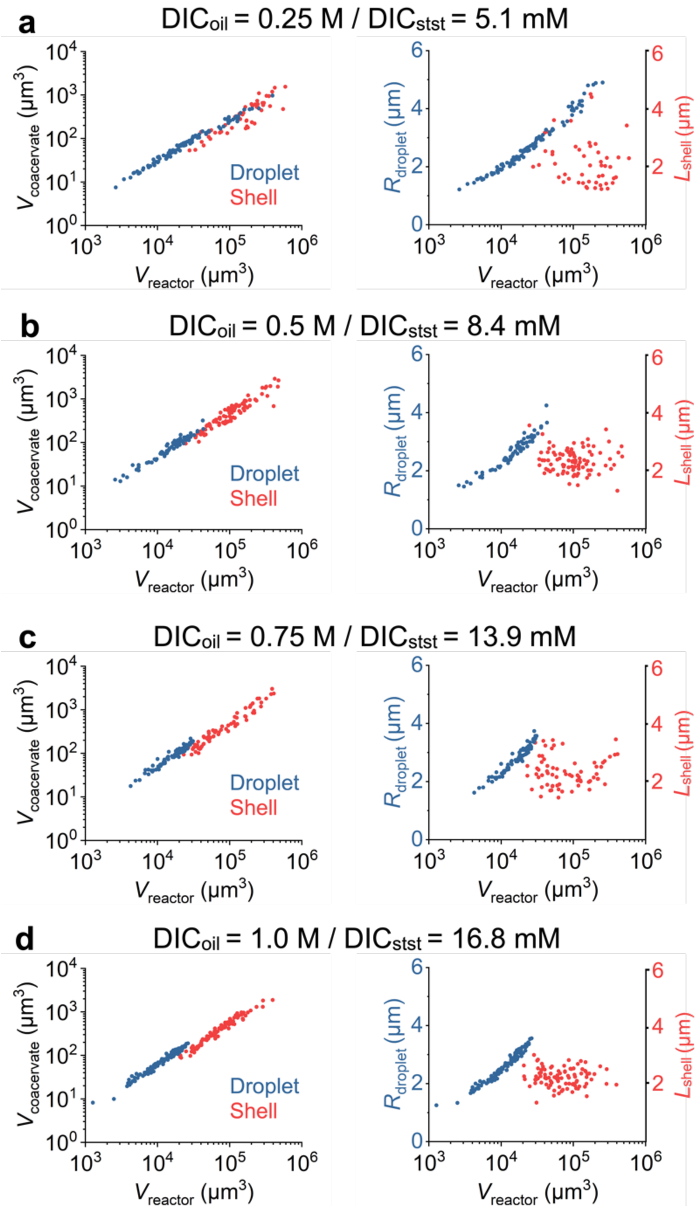

Supplementary Figure 8. Spherical shell formation with varying fuel concentrations. Conditions were 10 mM precursor, 5 mM pSS, and 200 mM MES at pH 5.3 with 0.1 μM sulforhodamine B. The indicated amount of DIC_{oil} was added to the oil phase to achieve a steady-state concentration of DIC_{stst} in the microfluidic reactors. After forming the spherical shells, active droplets and shells were measured at 0.5 h. All experiments were performed in triplicate (N=3). **a-d.** The volume of the total coacervate material as well as the radius R (blue) and shell thickness L (red) are shown for every individual microreactor that contained an active droplet (blue) or an active shell (red).

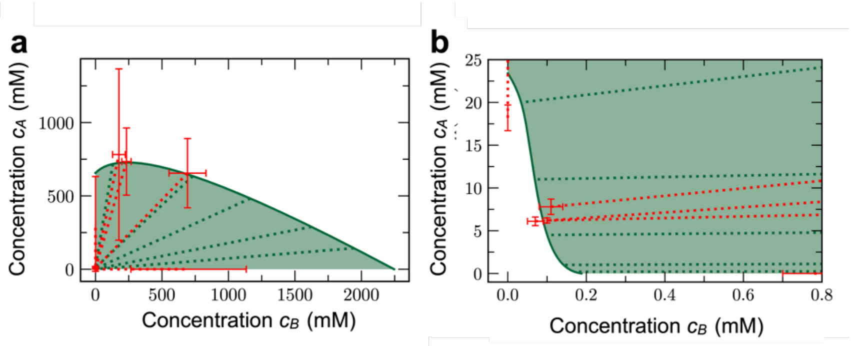

Supplementary Figure 9. Phase diagram. a-b. Five independent measurements (red data points with error bars) of different phase equilibria were used to fit the binodal line (solid green line) where two phases can coexist. The two corresponding concentration measurements are connected by a red dotted line, while the green dotted lines show a representative selection of tie lines of the model.

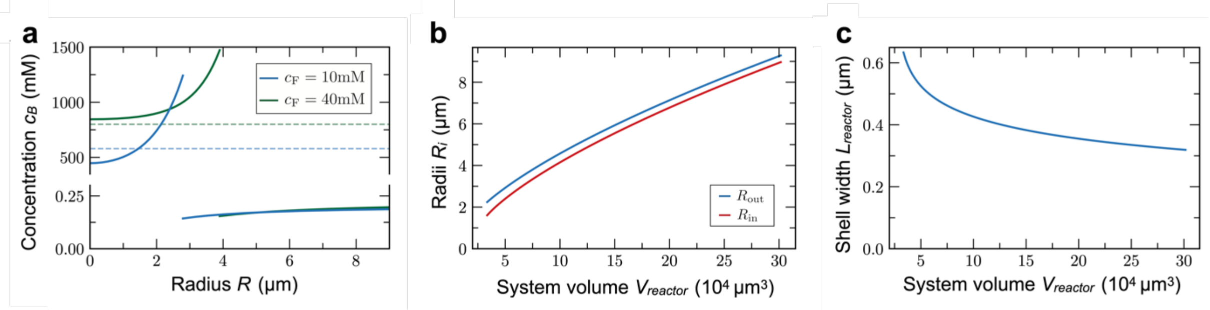

Supplementary Figure 10. Influence of different parameters on the spinodal instability at the droplet core and the shell width. a. Influence of fuel concentration on spinodal instability at the droplet's core. Radial concentration profiles for two different fuel concentrations are shown. For the lower fuel concentration (blue), the core of a droplet reaches the spinodal concentration (blue dashed). The stationary droplet is larger for the higher fuel concentration (green). However, the concentration at the core stays above the corresponding spinodal concentration (green dashed) due to weaker gradients resulting from a higher activation inside. For systems with high fuel concentrations, the precursor A gets activated and forms the product B inside the droplet. **b-c.** Influence of the system size on the shell width. Both interface radii increase as a function of system volume (**b**), but despite the 400 % increase of both radii, the distance between them $L_{\text{shell}} = R_{\text{out}} - R_{\text{in}}$ decreases only by 30 % (**c**).

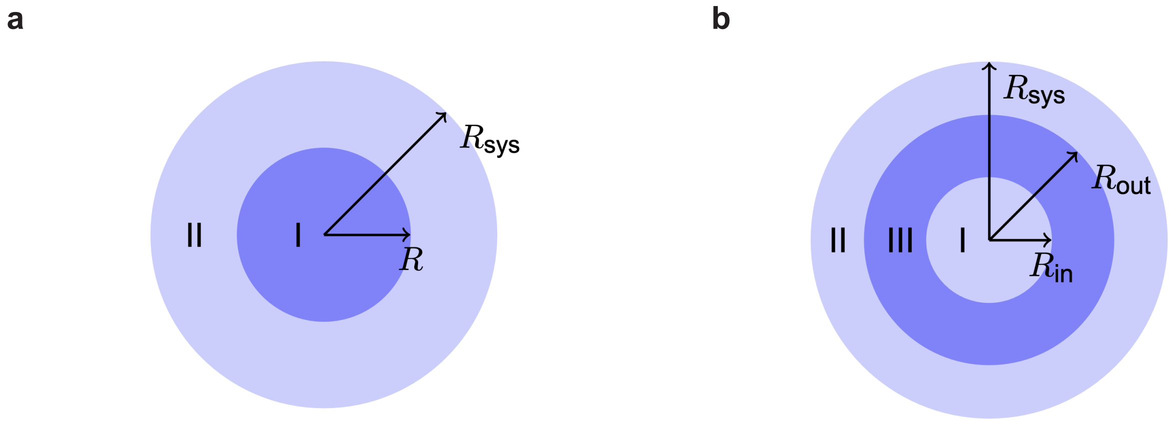

Supplementary Figure 11. Geometry for active droplet and active spherical shell. a. Geometry for active droplet. **b.** Geometry for active spherical shell.

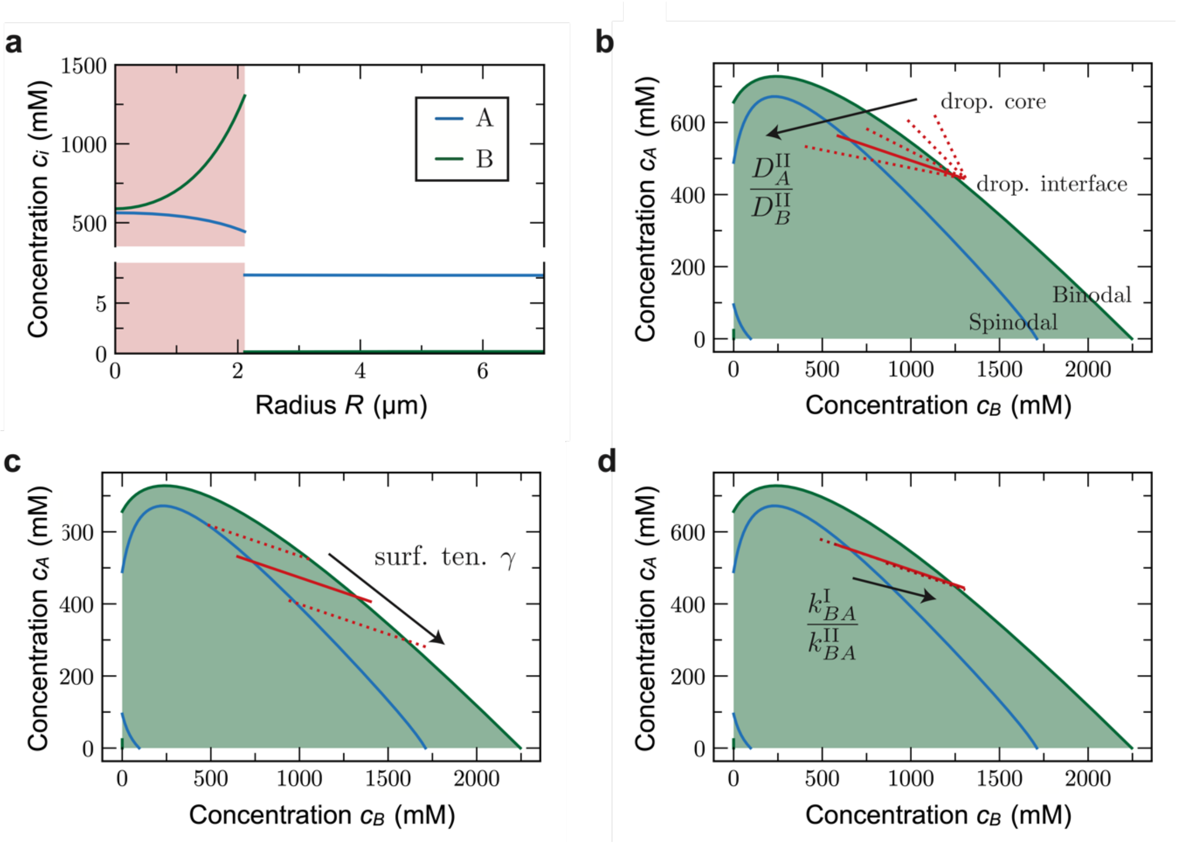

Supplementary Figure 12. Influence of different parameter choices. a. Concentration values of *A* and *B* as a function of the radial distance r from the droplet center (red shaded domain is the droplet inside). **b-d.** Concentration profiles of the components *A* and *B* of the droplet domain (red shaded domain in **a**) in composition space (red line) in addition to the binodal line (green) and the spinodal line (blue). Red solid line corresponds to the kinetic parameters used in the main text (Supplementary Tables 7 and 8).

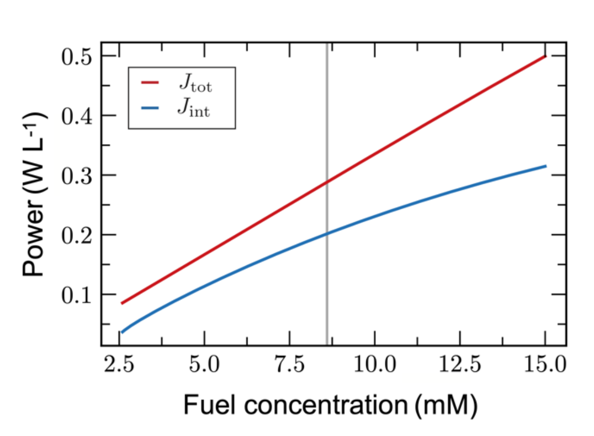

Supplementary Figure 13. Influence of fuel concentration on power turnover in the spherical shell state. The red curve represents the power per unit volume needed to constantly activate particles from state *A* to *B*, J_{tot}. We compare it to the free energy transported through the interface corresponding to activated *B* particles crossing the interface, J_{int} (blue curve). The vertical line corresponds to the fuel concentration value used for the estimates in the main text. In the shown fuel concentration range, the efficiency $J_{\mathrm{int}}/J_{\mathrm{tot}}$ varies non-monotonically between 46% and 70%.

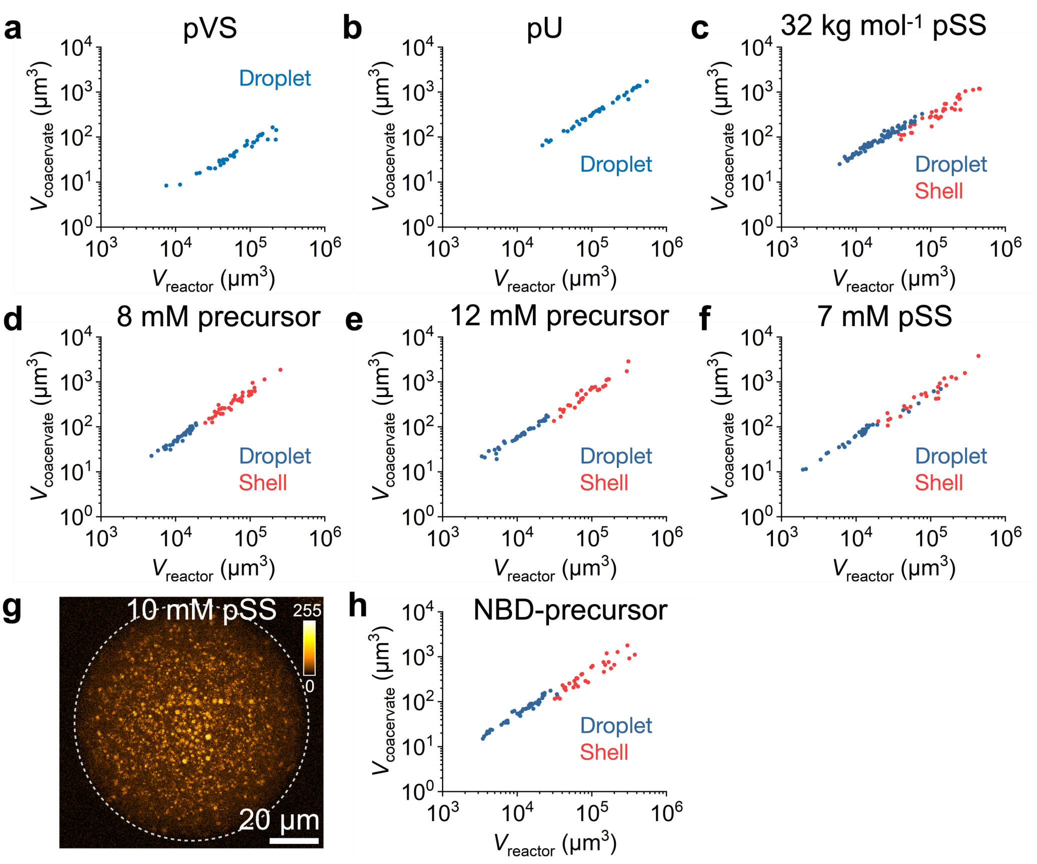

Supplementary Figure 14. Influence of various parameters on spherical shell formation. Standard conditions were 10 mM precursor, 5 mM pSS, and 200 mM MES at pH 5.3 with 0.1 µM sulforhodamine B, and only the indicated deviations from these conditions were applied. 0.5 M DIC was added to the oil phase to achieve a steady state. All experiments were performed in triplicate (*N*=3). **a-f.** The volume of the total coacervate material is shown for every individual microreactor that contained an active droplet (blue) or an active shell (red) upon a change in the indicated parameter. **g.** Maximum *Z*-projection of a representative microreactor 5 h after the induction of coacervation. Dotted circle represents the outline of the microreactor. The color scale is given next to the image. **h.** The volume of the total coacervate material is shown for every individual microreactor that contained an active droplet (blue) or an active shell (red) upon a change in the indicated parameter. NBD-G(RG)3-D-OH (NBD-precursor) was used as the dye.

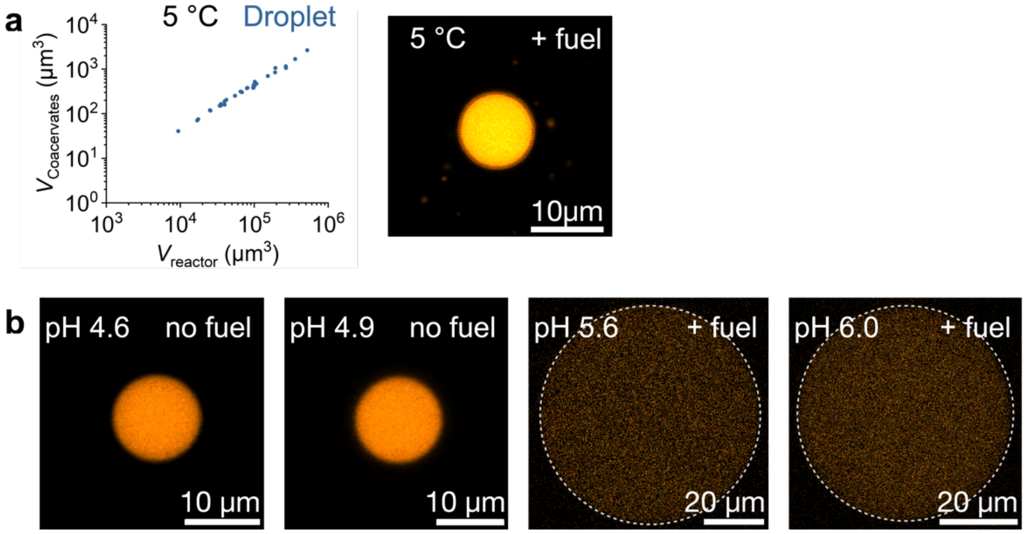

Supplementary Figure 15. Influence of temperature and pH on spherical shell formation. Unless indicated otherwise, conditions were 10 mM precursor, 5 mM pSS, and 200 mM MES at pH 5.3 with 0.1 µM sulforhodamine B. 0.5 M DIC was added to the oil phase to achieve a steady state. All experiments were performed in triplicate (*N*=3). **a.** The sample was kept at 5 °C for 3 h. The volume of the total coacervate material for every individual microreactor is plotted, and a representative image of a coacervate-based droplet shown to show that no transition into spherical shells independent of the volume was observed. **b.** MES buffer with indicated pH was used to prepare the samples. At pH 4.6 and 4.9, phase separation without the addition of fuel was observed. For pH 5.6 and 6.0, no phase separation was observed even after the addition of fuel. Dotted circles represent the outline of the microreactor.

V. Supplementary References

1. Jayalath, I.M., Wang, H., Mantel, G., Kariyawasam, L.S. & Hartley, C.S. Chemically Fueled Transient Geometry Changes in Diphenic Acids. *Organic Letters* **22**, 7567-7571 (2020).
2. Flory, P.J. Thermodynamics of High Polymer Solutions. *The Journal of Chemical Physics* **10**, 51-61 (1942).
3. Huggins, M.L. Thermodynamic Properties of Solutions of Long-Chain Compounds. *Transactions of the New York Academy of Sciences* **4**, 107-107 (1942).
4. Spruijt, E., Sprakel, J., Cohen Stuart, M.A. & van der Gucht, J. Interfacial tension between a complex coacervate phase and its coexisting aqueous phase. *Soft Matter* **6**, 172-178 (2010).
5. Brangwynne, C.P. et al. Germline P Granules Are Liquid Droplets That Localize by Controlled Dissolution/Condensation. *Science* **324**, 1729-1732 (2009).
6. Weber, C.A., Zwicker, D., Jülicher, F. & Lee, C.F. Physics of active emulsions. *Reports on Progress in Physics* **82**, 064601 (2019).
7. Bauermann, J., Weber, C.A. & Jülicher, F. Energy and Matter Supply for Active Droplets. *Annalen der Physik* **534**, 2200132 (2022).
8. Higuchi, T., Eberson, L. & McRae, J.D. Acid anhydride-free acid equilibria in water in some substituted succinic acid systems and their interaction with aniline. *Journal of the American Chemical Society* **89**, 3001-3004 (1967).
9. Miyamoto, S. & Shimono, K. Molecular Modeling to Estimate the Diffusion Coefficients of Drugs and Other Small Molecules. *Molecules* **25**(2020).
10. Donau, C., Spath, F., Stasi, M., Bergmann, A.M. & Boekhoven, J. Phase Transitions in Chemically Fueled, Multiphase Complex Coacervate Droplets. *Angewandte Chemie International Edition* **61**, e202211905 (2022).
11. Donau, C. et al. Active coacervate droplets as a model for membraneless organelles and protocells. *Nature Communications* **11**, 5167 (2020).
