# Supplementary material for: Liquid spherical shells are a non-equilibrium steady state of active droplets
Source: Nat Commun. 2023 Oct 17;14:6552. doi: 10.1038/s41467-023-42344-w (PMC10582082; doi:10.1038/s41467-023-42344-w)
Supplement: Supplementary file 4 — Description of Additional Supplementary Files [file 41467_2023_42344_MOESM4_ESM.pdf]

Description of Additional Supplementary Files

File Name: Supplementary Movie 1
Description: **Spherical shell formation upon dissolution.** Confocal micrograph timelapse series of a solution of 22 mM precursor, 5 mM pSS in 200 mM MES at pH 5.3 with 0.1 µM sulforhodamine B as a dye and fueled with 20 mM DIC. The active droplets grew and fused initially, and big droplets formed spherical shells upon dissolution. Imaging was done in PVA-coated ibidi chambers.

File Name: Supplementary Movie 2
Description: **Dissolution without spherical shell formation.** Confocal micrograph timelapse series of a solution of 22 mM precursor, 5 mM pSS in 200 mM MES at pH 5.3 with 0.1 µM sulforhodamine B as a dye and fueled with 10 mM DIC. The active droplets grew and fused initially but no spherical shells were formed upon dissolution. Imaging was done in PVA-coated ibidi chambers.

File Name: Supplementary Movie 3
Description: Formation of a stable active droplet. Confocal micrograph timelapse series of a solution of 10 mM precursor, 5 mM pSS in 200 mM MES at pH 5.3 with 0.1 µM sulforhodamine B as a dye. To achieve a steady state, 0.5 M DIC was added to the oil phase. The active droplets grew and fused until they reached a stable size of $r < r_{unstable}$. The time-lapse series represents the maximum Zprojection of a Z-stack throughout one microreactor. Imaging was started 10 min after the addition of fuel.

File Name: Supplementary Movie 4
Description: **Stable active droplet.** Confocal micrograph timelapse series of a solution of 10 mM precursor, 5 mM pSS in 200 mM MES at pH 5.3 with 0.1 µM sulforhodamine B as a dye. To achieve a steady state, 0.5 M DIC was added to the oil phase. The start of imaging was 2 h after the addition of fuel. A representative stable active droplet with $r < r_{unstable}$.

File Name: Supplementary Movie 5
Description: **Stable active shell.** Confocal micrograph timelapse series of a solution of 10 mM precursor, 5 mM pSS in 200 mM MES at pH 5.3 with 0.1 µM sulforhodamine B as a dye. To achieve a steady state, 0.5 M DIC was added to the oil phase. The start of imaging was 3 h after the addition of fuel. A representative stable active shell with $r > r_{unstable}$.

File Name: Supplementary Movie 6
Description: **Transition of an active droplet into an active spherical shell.** Confocal micrograph timelapse series of a solution of 10 mM precursor, 5 mM pSS in 200 mM MES at pH 5.3 with 0.1 µM sulforhodamine B as a dye. To achieve a steady state, 0.5 M DIC was added to the oil phase. An active droplet with a critical radius larger than $r_{unstable}$ transitioned into an active spherical shell. Imaging was done in a microreactor. Imaging was started 1 h after the addition of fuel.
